# Kiwifruit Metabolomics—An Investigation of within Orchard Metabolite Variability of Two Cultivars of *Actinidia chinensis*

**DOI:** 10.3390/metabo11090603

**Published:** 2021-09-06

**Authors:** Daryl Rowan, Helen Boldingh, Sarah Cordiner, Janine Cooney, Duncan Hedderley, Katrin Hewitt, Dwayne Jensen, Trisha Pereira, Tania Trower, Tony McGhie

**Affiliations:** 1 Fitzherbert Science Centre, The New Zealand Institute for Plant and Food Research Limited, Batchelar Road, Palmerston North 4410, New Zealand; sarah.cordiner@plantandfood.co.nz (S.C.); duncan.hedderley@plantandfood.co.nz (D.H.); tony.McGhie@plantandfood.co.nz (T.M.); 2 Ruakura Research Centre, The New Zealand Institute for Plant and Food Research Limited, Bisley Road, Hamilton 3214, New Zealand; helen.boldingh@plantandfood.co.nz (H.B.); janine.cooney@plantandfood.co.nz (J.C.); kati.hewitt@plantandfood.co.nz (K.H.); dwayne.jensen@plantandfood.co.nz (D.J.); trisha.pereira@plantandfood.co.nz (T.P.); tania.trower@plantandfood.co.nz (T.T.)

**Keywords:** metabolomics, variability, sampling, LC-MS, kiwifruit, *Actinidia chinensis*, ‘Hayward’, ‘Zesy002’

## Abstract

Plant metabolomics within field-based food production systems is challenging owing to environmental variability and the complex architecture and metabolic growth cycles of plants. Kiwifruit cultivars of *Actinidia chinensis* are vigorous perennial vines grown as clones in highly structured orchard environments, intensively managed to maximize fruit yield and quality. To understand the metabolic responses of vines to orchard management practices, we needed to better understand the various sources of metabolic variability encountered in the orchard. Triplicate composite leaf, internode and fruit (mature and immature) samples were collected from each of six *Actinidia chinensis* var. *deliciosa* ‘Hayward’ and *A. chinensis* var. *chinensis* ‘Zesy002’ kiwifruit vines at three times during the growing season and measured by LC-MS. In general, there was more variation in metabolite concentrations within vines than between vines, with ‘Hayward’ showing a greater percentage of within-vine variability than ‘Zesy002’ (c. 90 vs. 70% respectively). In specific tissues, the sampler, infection by *Pseudomonas syringae* var. *actinidiae* and the rootstock also influenced metabolite variability. A similar pattern of metabolic variability was observed from quantitative analysis of specific carbohydrates and phytohormones. High within-vine metabolic variability indicates that it is more important to obtain sufficient replicate samples than to sample from multiple vines. These data provide an objective basis for optimizing metabolite sampling strategies within kiwifruit orchards.

## 1. Introduction

Metabolomics can provide valuable insights into the biochemical processes underlying the developmental responses of plants [1]; however, to date metabolomics research has largely focused on the responses of individual plants [2]. Applied to populations of plants, the emphasis has been on the development of markers to assist in molecular breeding [2]; understanding major issues in agronomy such as drought acclimation, and responses to salinity [3,4]; or the biochemistry underlying the quality and postharvest behaviour of important plant products such as fruits [5]. There has been less emphasis on using metabolomics to understand the responses of field-grown perennial or woody plants to in-field manipulations aimed at improving plant health, productivity or responses to environmental stressors.

The translation of plant metabolomics research from model species grown under controlled environment conditions to field, orchard or forest plant production systems faces a number of challenges, both analytical [6] and arising from the uncontrolled field environment and the real-time diurnal and seasonal growth cycles of perennial, annual and woody plants. Experimental designs for the metabolomics of forest trees [7] including birch (*Betula pendula* Roth) [8], poplar (*Populus balsamifera*) [9], pine (*Pinus radiata* and *P. pinaster*) [10,11] and for the comparison of the foliar metabolomes of tropical trees [12] have been described. In contrast, orchards and vineyards offer more intensively managed environments and commonly feature regular repeated arrangements of clonal plants groomed and trained onto permanent trellis or pergola structures and specifically orientated with respect to the sun and the local topography. Each orchard, vineyard (or equivalent) is, however, located in a specific environment and plant performance is dependent on a site-specific history of cultivation, fertilization, and management. Within the fabric of the orchard itself, individual plants, leaves, stems, and branches experience different environments, resulting in opportunities for differing metabolic responses, and experimental and analytical challenges [6] of obtaining representative metabolomics data. Orchards and field-based plant production systems are also real time, and often high-value, food production systems, which may limit the opportunities for experimental manipulation and the collection of samples.

The importance of appropriate sample collection, processing and analysis protocols when working with new, variable or multiple tissue types has been repeatedly emphasized [6,7,13]. A number of sampling strategies have been reported for obtaining representative samples of grapes from vineyards for metabolite analyses. In an analysis of the effect of vine vigour on skin procyanidin composition of *Vitis vinifera* L. Pinot noir growing in a commercial vineyard of the same clone, rootstock, age and management practices, triplicate samples consisting of 150 berries each were used [14]. Within-vineyard variability of the pepper flavouring sesquiterpene rotundone was shown to relate to the land underlying the vineyard [15] using 100-g subsamples obtained from three bunches. In an experiment set up to favour genetic determinism, LC-MS based metabolomics differentiated eight *V. vinifera* grape varieties based on stem polyphenolics, with metabolic distance between cultivars related to genetic distance based on microsatellite DNA markers [16]. To overcome intra-plot variability, a randomized draw was used constitute each of the five pseudo-biological replicates analyzed. In a metabolomic investigation of *terroir* using a single clone of Corvina in seven vineyards over three years [17], 30 grape clusters were randomly collected along two vine rows, three berries were selected from each cluster, and subsamples used to make a ten-berry pool for chemical analysis. While successful in enabling differentiation of experimental treatments, the rationales for the above sampling regimes are not explicit. To enable metabolomics analysis in such situations, obvious responses are to increase environmental control or effect size differences through experimental manipulation; to increase the sampling rate; or to invoke ‘big data’ and the use of more sophisticated data analysis methods. An alternative approach is to seek to a better understanding of the sources of metabolic variability, resulting in more effective experimental design and sample collection strategies.

Kiwifruit, cultivars of *Actinidia chinensis* (Planch) *and A. arguta* (Planch), are vigorous dioecious perennial vines, originally from eastern Asia, and now cultivated worldwide for their edible fruit [18]. Commonly grown New Zealand cultivars include the green-fleshed cultivar *A. chinensis* var. *deliciosa* ‘Hayward’ and the yellow-fleshed cultivar *A*. *chinensis* var. *chinensis* ‘Zesy002’. Kiwifruit are commonly grown as clonal material grafted onto clonal or seedling rootstocks and trained onto horizontal pergola structures. Vines are managed intensively to balance fruit yield and quality [19], vegetative growth [20] and year-on-year productivity [21,22]. Typical on-orchard management practices include reduction of excessive vegetative growth, thinning of leaves (Thorp 2003) and girdling of the trunk, or root pruning, to temporarily cut the phloem and redirect the flow of photosynthate from the leaves to the fruit rather than to the roots [23,24]. Kiwifruit vines are also susceptible to bacterial canker arising from infection with the pathogenic bacterium *Pseudomonas syringae* pv. *actinidiae* (Psa) [25]. Infections can be managed though on-orchard cultural practices [26], biocontrol agents such as endophytic bacteria [27], or by the use of synthetic elicitors such as Actigard^™^ (acibenzolar-S-methyl, ASM), which enhance natural resistance pathways and modulate the metabolism of the vines [28].

Understanding how these chemical and physiological tools affect the metabolism and performance of kiwifruit vines requires the development of new experimental protocols and a better understanding of the sources of metabolic variability (within plant and within orchard), the dynamics of metabolite responses (appropriate sampling times and tissues), and of the key metabolites involved. Surprisingly, between-vine variation accounted for only a minor part of the total variability in fruit dry weight, soluble solids and firmness for ‘Hayward’ fruit harvested from two different pergola systems, describing only 0% or 2.2%, 13.8% or 19.0% and 7.1% or 15.5% of the total variance respectively [29]. Variability within ‘zones’ of the vine canopy itself made significant contributions to the total variance for these parameters [30]. Information on the extent and sources of variability of individual plant metabolites is not readily available from the kiwifruit metabolomics literature [31,32,33,34,35,36,37].

In this paper we describe the metabolomics analysis of mature and immature leaf, fruit, and internode shoot samples collected from multiple canes on multiple ‘Hayward’ and ‘Zesy002’ kiwifruit vines at three physiologically significant time points throughout the growing season. We identify sources of metabolic variability including the contribution of between-vine variation, sampler, Psa infection and rootstock effects, and discuss the implications for the design of metabolomics experiments to better understand changes in plant metabolism in response to orchard manipulations to improve plant performance.

## 2. Results

**Figure 1:**
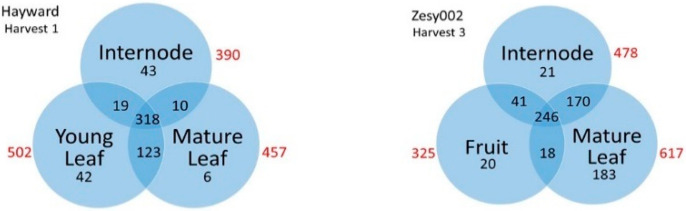
Venn diagrams showing the distribution of metabolites among different tissues of ‘Hayward’ (Harvest 1) and ‘Zesy002’ (Harvest 3) kiwifruit vines. Data are presented for 583 and 699 metabolites having greater than 80% and 100% detection thresholds in Hayward and ‘Zesy002’ kiwifruit respectively as measured in bulked QC samples. Red numbers are the total number of metabolites detected in that tissue.

The LC-MS metabolomics method used here is representative of methods commonly used in plant metabolomics and measures c. 170–600 metabolites (mass tags): sugars, polyphenolics, triterpenes, complex lipids and other metabolites in fruit, leaf and internode tissues of ‘Hayward’ and ‘Zesy002’ kiwifruit. The majority of metabolites measured were common to all tissues (Figure 1) and while many metabolites showed cultivar, tissue and harvest-specific differences, only a few were specific to particular tissues or to one cultivar. Thus for ‘Hayward’ at Harvest 1, 76% metabolites (441) were shared between young and mature leaves and a majority of metabolites (55%, 318) were shared among all three vegetative tissues. Similarly, for ‘Zesy002’ at Harvest 3, 60% of metabolites (416) were shared between leaf and internode tissues, and a substantial proportion (35%) were found in leaf, internode and fruit. 

Analysis of the composite QC samples indicated that 90–99% of the metabolites in leaf and shoot internode tissues, and c. 84% of metabolites in fruit samples, were measured with coefficients of variation (CVs) of less than 20% (Supplementary Table S1). Analysis of technical replicates, duplicate samples prepared from the same sample of frozen or freeze-dried tissue, showed a loss of precision compared with the QC composite samples, in particular for the ‘Hayward’ leaf and internode samples at Harvest 1 (Table 1). Extraction of a single leaf sample (50, 100 and 200 mg DW, ‘Zesy002’ Harvest 3) with the same ratio of extraction solvent did not demonstrate any changes in method precision that might arise from sample inhomogeneity. For the comparison of experimental treatments, a CV of 20% allows a reasonable balance of required sample numbers with anticipated effect sizes [38]. Overall, 66–98% of all the metabolites measured in these samples showed a CV of less than 20%. The median CVs for metabolites in all samples ranged between 5.7 to 13.1% (mean 8.9%), considerably less than that expected and subsequently observed for plant samples collected in triplicate from multiple vines.

In previous (unpublished) kiwifruit experiments we have considered a 1.2- to 1.5-fold change in metabolite concentrations as being of interest. A power calculation suggests that to measure a 1.5- or 1.2-fold change in the concentration of a metabolite having a CV of 20% (with 80% chance of *p* < 0.05), 4–17 samples respectively per treatment are required. This is the number of samples which might reasonably be collected over a short time period (1–2 h) by one person working in an orchard. The metabolomics methodology is therefore expected to be able to measure 1.2- to 1.5-fold treatment differences for several hundred metabolites in orchard-collected samples.

### 2.1. Between- and Within-Vine Variability in Metabolite Concentrations and Metabolite Selection

**Figure 2:**
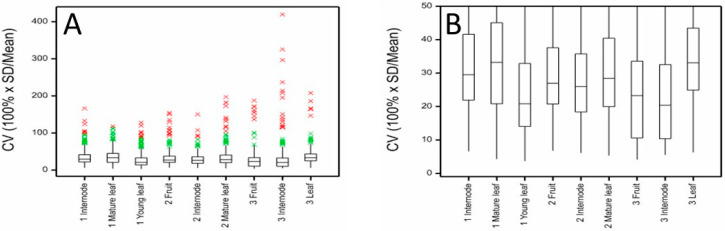
Distribution of coefficients of variation (CV) for metabolites measured by LC-MS in internode, mature and young leaf, and fruit of ‘Hayward’ kiwifruit samples showing (**A**) the full range of values and (**B**) the interquartile ranges. The boxes cover the middle 50% of measurements; 25% are below the bottom of the box and 25% are above. The whiskers cover no more than 1.5× height of box above the top or below the bottom of the box. Beyond that, individual observations are shown in green (1.5× height of box to 3 × height of box) or red (more than 3× height of box beyond the box).

**Figure 3:**
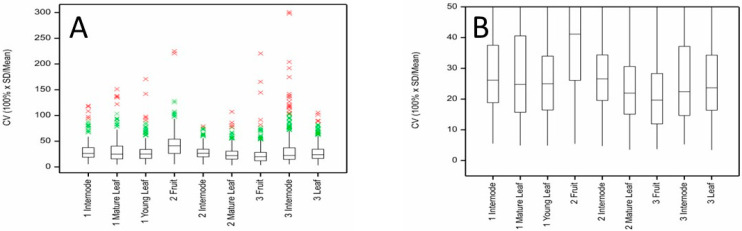
Distributions of coefficients of variation (CV) for metabolites measured by LC-MS in ‘Zesy002’ kiwifruit internode, mature and young leaf and fruit tissues at three harvest times showing (**A**) the full range of CV values and (**B**) the interquartile ranges.

Box-and-whisker plots were used to examine the range of CVs encountered for all the metabolites measured in each ‘Hayward’ (Figure 2) and ‘Zesy002’ (Figure 3) kiwifruit tissue. While a few metabolites have very high CVs, most were below 40% with a mean in the range 20–30%. ‘Zesy002’ fruit measured at Harvest 3 were especially variable, with a mean CV of c. 40%.

Random effects models were used to calculate the between- and within-vine components of variance for each metabolite for each tissue and sampling time, and what percentage of the total variability between samples was due to between-vine (inter-vine) variation. 

**Figure 4:**
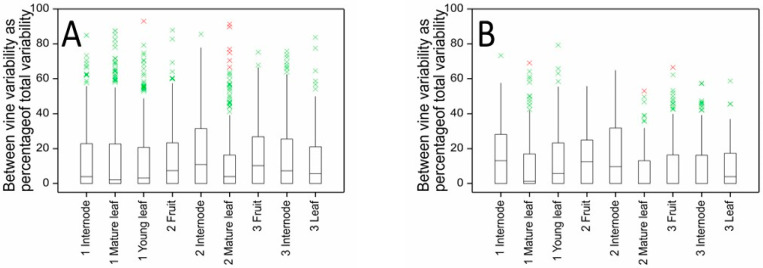
Between-vine (inter-vine) variability as a percentage of total variability for kiwifruit metabolites measured in fruit, internode and leaf tissues of ‘Hayward’ kiwifruit by LC-MS at three harvest times showing the distributions of percentage variance for (**A**) all metabolites and (**B**) for only those metabolites with CV < 20%.

**Figure 5:**
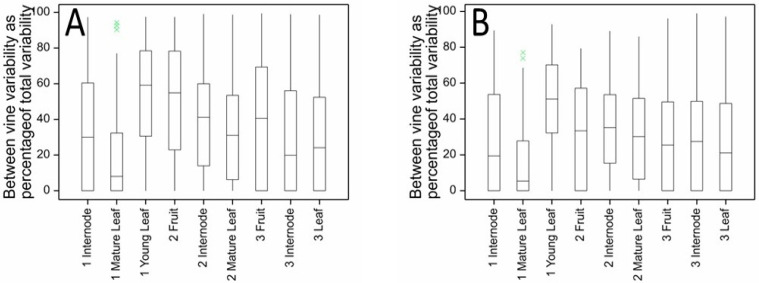
Between-vine (inter-vine) variability as a percentage of total variability for kiwifruit metabolites measured in fruit, internode and leaf tissues of ‘Zesy002’ kiwifruit by LC-MS at three harvest times showing distributions of percentage variances for (**A**) all metabolites and (**B**) for only those metabolites with CV < 20%.

The distribution of between-vine variability for kiwifruit metabolites (measured as a percentage of total variability) in fruit, internode and leaf tissues of ‘Hayward’ kiwifruit is shown in Figure 4A. In all tissues and at all harvest times, the median percentage variability due to between-vine variation was very low, less than c. 10%. This means that for most metabolites there was more variability in metabolite concentrations occurring between samples taken from the same vine than was found when comparing samples taken from different vines. For ‘Zesy002’ (Figure 5A), the median percentage of variability explained by between-vine variability ranged from less than 10% for mature leaves at Harvest 1 to c. 60% for young leaves also collected at Harvest 1. The median value was about 30%. The higher percentage of between-vine variability observed for ‘Zesy002’ compared with ‘Hayward’ (mean between-vine variability c. 10%) may reflect the higher incidence of Psa infection observed within this experiment, or the presence of two different rootstocks bearing the ‘Zesy002’ vines.

The higher between-vine variability and the more variable influence of inter-vine effects observed with ‘Zesy002’ samples necessitates different and more specific sampling strategies than might be used with ‘Hayward’ kiwifruit. High between-vine variability (mean of 60%) for young leaves collected at Harvest 1 indicates significant differences in the metabolite concentrations between different vines and the importance of collecting samples from multiple vines. The low between-vine variability (mean c. 10%) for mature ‘Zesy002’ leaves also collected at Harvest 1 indicates that the differences in metabolite concentrations observed within mature leaves on an individual vine are generally greater than those observed between vines—one might, all factors being equal, just collect samples from one vine.

For ‘Hayward’, many of the metabolites with a high-percentage between-vine variability (>60%) also had CV > 20%. Removal of metabolites with CV > 20% (Figure 4B) resulted in a minor reduction in the percentage of between-vine variability. For ‘Zesy002’ and considering only the metabolite data where the CV was <20% (Figure 5B), the distribution of between- versus within-vine variability was about the same as for the complete dataset; however, the proportion of between-vine variability was generally reduced. For example, for internode and young leaf metabolites at Harvest 1, the percentage variance explained by between-vine variation decreased from c. 30% and 60% to c. 20% and 50%, respectively.

The removal of metabolites with CV > 20% resulted in a set of metabolites more coherent with a sampling strategy based on low between-vine variance. Restricting the data analysis to metabolites with a CV of 20% or less also enables the collection of a practical number of samples for statistical analysis, as discussed above. In practice, twenty samples per treatment seems a reasonable maximum number to be collected, given the number of vines that might be available, and the time taken for one person to collect these samples over a reasonable diurnal time period (1–2 h) on any one day. For these reasons, all metabolites with CV > 20% were excluded from subsequent statistical analyses. The numbers of such metabolites measured in ‘Hayward’ and ‘Zesy002’ internode, young and mature leaf tissue and fruit are given in Table 2.

### 2.2. Effects of Sampler, PSA Infection, and Rootstock on Metabolite Concentrations and Between-Vine Variability

We next investigated in more depth the contribution of the sampler, the presence of Psa infection in the vine and of the rootstock to the variances observed in the concentration of metabolites in experimental samples. Restricting the data analysis to only those metabolites that showed a CV < 20%, and for which we could realistically measure treatment effects, reduced the number of metabolites to about one third; however, we still had measurements on relatively large numbers of metabolites (Table 2). Sampler effects were suspected from previous work, and while the incidence of Psa symptoms and infection of vines was initially low, it increased during the second year, resulting in the loss of samples from some ‘Zesy002’ vines. The discovery that the ‘Zesy002’ vines were either doubly grafted, grafted onto a ‘Hort16A inter-scion on ‘Bruno’ rootstock, or grafted directly onto ‘Bruno’ rootstocks, was considered as a not-unusual occurrence in an orchard, and provided an opportunity to test the sensitivity of our analytical methods. 

The effects of sampler, Psa infection (visual symptoms yes or no) and rootstock (for ‘Zesy002’) were tested by fitting these parameters as fixed effects in the mixed effects model used to calculate within-vine and between-vine components of variance and seeing whether removing these parameters made the model significantly worse. Here significance was set at α = 0.01 to reduce the number of false positives. Alternatively, Adonis (R vegan package, [39]), a multivariate ANOVA-like technique developed for use in ecology to analyse differences in community composition in response to the environment, was used to analyse for effects on metabolites due to vine-to-vine (inter-vine) variation, sampler, Psa symptoms and rootstock. Adonis is similar to multivariate analysis of variance (MANOVA) and uses a permutational multivariate analysis of variance to partition distance matrices among sources of variation. For example, Adonis might be used to analyse differences in numbers of species present (metabolite concentrations) at multiple ecological sites (individual vines) in response to different treatments (presence of Psa).

In ‘Hayward’ kiwifruit there appeared to be significant Psa, sampler and inter-vine effects for young leaves, and significant inter-vine effects for mature leaves when collected at Harvest 1 (Table 3). Metabolite concentrations in young (Harvest 1) leaves seemed particularly sensitive to the presence of Psa infection and to sampler bias, with the concentrations of 4 and 12% of metabolites being significantly affected by the presence of Psa and the sampler, respectively. Young leaves may be more affected by Psa than other tissues, as the tissue is softer, and it may be easier for the bacterium to enter. Alternatively, if the plant is stressed in responding to Psa infection, then young leaves as metabolite sinks may be especially affected. Mature and young leaves from Harvest 1 were also the only tissues where significant numbers of metabolites (7%) showed significant between-vine differences, consistent with the presence of Psa infection in some vines.

In all other tissues, the number of metabolites affected by the presence of Psa symptoms or sampler or showing significant inter-vine differences was not greater than might be expected by chance, supporting the contention that in general there is more variability in metabolite concentrations within than between vines (Figure 4 and Figure 5). Only for Harvest 1 leaf tissues would it be it important to sample from multiple vines, and even here the majority of metabolites did not show significant inter-vine effects.

Analysis of ‘Hayward’ datasets using the Adonis procedure gave somewhat different conclusions (Table 3). The effect of Psa symptoms on vegetative tissues was not significant and nor were sampler and inter-vine effects except in the case of fruit and young leaf (sampler effects) and for fruit and internode tissue at Harvest 3 (between-vine effects). To assess these results, we considered the PCA plots for all metabolites with CVs less than 20% on which this analysis was based (Supplementary Materials). For Harvest 1, the sampler effect in young leaves (*p* = 0.005) would seem to arise from differences in variability between the samplers evident in the PCA plot. For Harvest 2, the sampler effect Fruit (*p* = 0.001) corresponded to discernible differences in group means in PC1, whereas the difference (*p* = 0.04) for Harvest 3 fruit may again arise more from the greater variability of samples collected by one of the two samplers. Inter-vine differences for fruit and internode tissues collected in Harvest 3 can be rationalized from the PCA plots, at least for the internode tissue (*p* = 0.007).

Overall, for ‘Hayward’, the occurrence of Psa, sampler and inter-vine effects affecting the concentrations of significant numbers of metabolites (more than might be expected by chance) was the exception rather than the rule. ANOVA and Adonis approaches to data analysis gave different results, implying that these effects are “subtle” even if affecting many metabolites. Interestingly, inspection of the PCA plots suggests the sampler effects identified by Adonis result from differences in variability between samplers rather than any differences in mean metabolite concentrations. Particularly interesting is the general absence of between-vine effects i.e., within-vine variation was generally much larger than between-vine variation. There are still very good reasons to sample multiple vines, but this result provides license to sample intensively within vines in order to make up sample numbers when vine numbers are limited.

For ‘Zesy002’, the number of metabolites showing Psa effects and sampler effects were within the range expected by chance (Table 4). There were, however, a significant number of metabolites affected by between-vine differences for immature fruit at Harvest 2 and by rootstock effects for internode and leaves at Harvest 2, and for fruit and internode at Harvest 3. In total, four out of nine tissue types showed significant numbers of metabolites affected by the type of rootstock present. Testing for the influence of rootstock on metabolic variability was not part of the original design of this experiment. However, as a result of historical management of the orchard, two different rootstocks ‘Bruno’ seedling rootstocks and ‘Bruno’ rootstocks with a ‘Hort16A’ inter-scion [40] were present located in separate rows. The orchard is located on a north-facing (south to north) slope with the vines growing in rows up and down the slope. The position of vines on the slope within a row, rather than different rows, is known to be the major source of variation in the development of these vines, for example, the timing of budbreak and fruit ripening. For this reason, we consider these differences in metabolites to result from the presence of different rootstocks, but this should be confirmed in further experiments.

Testing the datasets with the multivariate Adonis procedure again gave somewhat different results with Psa infection and rootstock at Harvest 2 as the most significant effects (Table 4). These conclusions were again validated by inspection of the relevant PCA plots (Supplementary Materials). For Harvest 1, no significant effects were found and the PCA plots showed insufficient separations to define treatments groups properly. For Harvest 2, Adonis gave an effect of Psa symptoms (*p* < 0.02) for fruit and internode tissues. For fruit, this result appears based on three out of five ‘outlying’ points in PC1; for internode tissue, separation of samples was visible along PC1. The rootstock effect in internode and mature leaf tissue (*p* = 0.003 and 0.001 respectively) also appears as an obvious separation of clusters along PC1. For Harvest 3, the sampler effect for fruit (*p* = 0.02) identified by Adonis appears to arise from differences both in the variability (spread) of the two groups and the alignment on PC2. Interestingly, the Harvest 3 internode samples (significantly different by ANOVA) also showed differences between groups in the PCA plot (but *p* = 0.06). Generally, an Adonis effect with *p* < 0.02 was discernible as differences in group variances or separation in the PCA plots, providing some validation for the results of the Adonis analysis. 

The Adonis procedure proved useful in putting an objective value to observable differences in the PCA plots. Adonis does not assume equal variances between treatments. This may account for differences in the outputs from the Adonis and ANOVA analyses.

### 2.3. Targeted Analyzed of Soluble Sugars and Phytohormones

We were interested to see if the high within-vine variability encountered in our metabolomics analysis also occurred when metabolites were measured using targeted analytical protocols. Two classes of metabolites were examined, soluble sugars and polyols (glucose, fructose, sucrose, planteose, myo-inositol and galactinol) present at mg/g concentrations, and nine phytohormones, selected for their co-occurrence in multiple tissues and present at ng/g concentrations. The measured concentrations of these metabolites (LOQ defined as >5 times signal/noise) and their variances are given in Supplementary Tables S2–S4. 

Metabolites measured by targeted analysis (Table 5), showed wide differences in variability but with a distribution of variances comparable to those observed for metabolites measured using untargeted metabolomics (Figure 1 and Figure 2). The percentage CVs for technical replicates (Supplementary Tables S2–S4) were less than 10% (‘Hayward’ mean %CV 6.4, ‘Zesy002’ mean %CV 5.2), confirming that the observed metabolite concentration variance is principally due to biological variability. The phytohormone measurements showed a somewhat higher variability, reflecting the responsiveness of these metabolites to the orchard environment, where plants are subject to considerable variations in light intensity, temperature, air and soil moisture, and soil composition. For this reason, phytohormones are commonly measured using in vitro-grown plant materials [41]. The mean percentage of variability (CV) due to between-vine variability was also highly variable between individual metabolites, but again comparable to those observed for metabolomics data (Figure 2 and Figure 3). For both the soluble carbohydrates and phytohormone metabolites, ‘Hayward’ again showed a lower percentage of variability due to between-vine variation than ‘Zesy002’. These results extend the earlier observations of high within-vine, and low between-vine variability, for fruit dry matter, soluble solids and firmness reported by for ‘Hayward’ kiwifruit fruit [29,30]. This pattern of high within-vine metabolic variability, observed for differing sets of metabolites measured by different analytical methods, suggests that this variability is intrinsic to kiwifruit metabolism and must be considered in transitioning metabolomics analysis into the orchard.

## 3. Discussion

While metabolomics can routinely measure the relative concentrations of hundreds of metabolites in biological samples, there has been limited progress in applying this technology to understanding the metabolic responses of complex biological systems such as perennial plants growing in fields or orchards. While control of environmental variability and better analytical technology may reduce metabolic variability, there are also practical limitations on the availability of materials and the number of samples that can be subjected to chemical analysis. An additional strategy is to seek to a better understanding of the sources of metabolic variability, resulting in better experimental design, more effective data collection and greater chances of observing meaningful metabolic changes.

From the metabolic variability measured in this study, we can propose some practical considerations to guide experimental design and the collection of field samples. The variability of metabolite concentrations (CV) defines how many samples are needed in order to reasonably expect to see significant differences between experimental treatments, if such exist. Power calculations conservatively estimate 20 samples per treatment are required to meaningfully measure a 1.2-fold change in metabolite concentrations with a CV of 20%. If we anticipate measuring only higher (1.5-fold) fold changes in metabolite concentrations, then smaller sample numbers are sufficient. These sample numbers, coupled with the need to consider diurnal and daily differences in metabolite concentrations, limit the time available for sample collection and the number of experimental treatments that can readily be sampled in one day from a field experiment.

The balance of between- (inter) and within- (intra) vine variability determines the best balance between the number of vines to sample and the number of replicate samples to be taken within any vine. For example, for metabolites with high within-vine variability there is little advantage or necessity for sampling from additional vines. Our analysis indicates that, generally, there is more variability in metabolite concentrations occurring within individual than between different vines. However, between-vine variability of metabolites was not zero, and differed between cultivars and tissues, and along with Psa, rootstock, sampler and time of collection, will contribute to the total sample variability. The relative importance of between- and within-vine metabolite variability may require (or permit) different sampling strategies in particular for ‘Hayward’ (inter-vine variability c. 10%) where there is little advantage, overall, in sampling from more than one vine. Sampling from only one vine, however, provides no reassurance to the researcher that that vine is representative, or insurance against random events such as vine death due to Psa infection. At a practical level, the number of samples that can be collected from one vine (for example, a sufficient number of newly expanded first leaves) may necessitate sampling from multiple vines especially if repeated sampling of the same vine is anticipated. The number of different samplers required to collect samples and whether sampling be completed in a reasonable time, without confounding effects due to diurnal fluctuations in metabolite concentrations, also needs to be considered. This suggests despite the low inter-vine variability, the best sampling strategy to fairly represent metabolite variability remains to sample from as many vines as possible but more importantly to sample within the available vines aiming primarily for sufficient biological replicates.

The increasing incidence of Psa infection and the belated discovery that the ‘Zesy002’ vines were grafted onto two different rootstock types are examples of the constraints imposed when working in real-world environments and of the requirement for robust experimental design, ideally oversampling in data collection and flexibility in data analysis. In the event, these events enabled us to test the sensitivity of our analytical methods and demonstrated additional factors affecting metabolite variability. Specific effects on individual metabolites are not easily distinguishable from statistical noise in metabolomics experiments and while statistical analysis provides evidence that most often Psa, sampler and rootstock effects were not statistically significant, they may still contribute to overall metabolite variability. Such effects might be more sensitively probed at the biochemical pathway level, using metabolite identification to look for coordinated responses in individual pathways.

A limitation for practical application of this study was the relatively low proportion of metabolites (c. 20%) for which confident or tentative identifications based on accurate mass and MS/MS analysis of isomeric compounds can be made [42]. Actinidia is a relatively unstudied genus, and while the nutrient composition of the fruit of commercial cultivars is well known [43], Actinidia species contain multiple other metabolites including polyphenols and their glycosides, polyhydroxylated triterpenes, carotenoids [44,45], procyanidins [46] and novel norterpenoids [47] and nitrogen-containing flavanols [48]. A more comprehensive identification of metabolites will be required [49] to understand the pathway-level responses of these plants to developmental and environmental changes, and this is a priority for future research [50].

In conclusion, metabolites in multiple kiwifruit tissue samples were measured by untargeted and targeted LC-MS metabolomics with sufficient instrumental precision to measure the relative contributions of within- and between-vine variances to metabolite variability, and to demonstrate metabolic effects due to Psa infection, the sampler and the rootstock. In general, there was more variation in metabolite concentrations within vines than between vines. High within-vine metabolic variability indicates the important of obtaining sufficient replicate samples rather than sampling from multiple vines. These results provide the basis for future research to understand the effects of elicitors and other protectant chemicals, of rootstocks, and of other on-orchard practices, on kiwifruit vine metabolism and performance.

## 4. Materials and Methods

Actinidia chinensis Planch. var. deliciosa ‘Hayward’ and A. chinensis Planch. var. chinensis ‘Zesy002’ kiwifruit vines were sampled during the 2016/17 and 2017/18 growing seasons respectively from plants growing at the Plant and Food Research, Ruakura Research Orchard, Hamilton, New Zealand. The orchard is located at 37°46′20.5″ S 175°18′52.7″ E, with opposing male vines for pollination and rows running approximately north-south on a north facing slope. Canes (5–20 mm thick at the base and which had held fruit the previous year) were tagged and each paired with a cane from the opposite side of the vine, resulting in one to three paired canes per vine. Samples were collected from shoots on each of two paired canes as: young leaves, five young leaves just unfurled with mid rib removed; mature leaves, youngest mature leaf associated with a flower or fruit; internode tissue, the shoot internode above and below the youngest mature leaf (excluding the leaf node tissue); and fruit, one fruit per cane. At Harvest 3, a 1/8th longitudinal slice was collected from each fruit.

For the 2016/17 (southern hemisphere) season, triplicate samples of mature and young leaves, developing fruit, and shoot internodes were collected once from multiple canes from each of 10 ‘Hayward’ vines grafted onto ‘Bruno’ rootstock (Table 6). These samples were collected at king flower calyx split (14 November, Harvest 1, H1), during rapid fruit growth (20 December, Harvest 2, H2) and during peak starch accumulation (20 February, Harvest 3, H3). In 2017–2018, samples were collected from four ‘Zesy002’ vines grafted onto ‘Bruno’ rootstock (row 14) and from ten ‘Zesy002’ vines stump-grafted to ‘Bruno’ rootstock with a ‘Hort16A’ (A. chinensis var. chinensis) inter-scion (rows 17 and 18) [40]. Triplicate samples of mature and young leaves, developing fruit, and shoot internodes were collected once from multiple canes at king flower calyx split (6 November, H1), during rapid fruit growth (15 December, H2) and during peak starch accumulation (15 February, H3). Observations of any Psa symptoms such as spotting on leaves, the person collecting the samples (the sampler) and, for ‘Zesy002’ vines, the rootstock type of individual vines were recorded. 

Samples were immediately frozen in liquid nitrogen, combined with the paired sample from the same vine, and stored at −80 °C. All sample types except fruit were freeze-dried before grinding and extraction. Fruit samples were ground while frozen with liquid nitrogen. Dry matter, starch content, and a range of phytohormones and soluble carbohydrates were also measured [51,52]. All samples were collected between 1030 and 1300 h on a single day. Our objective was to collect a minimum of three replicate samples from each of six vines. In the event, more ‘Hayward’ vines were available, but not all vines had sufficient canes necessary to permit the collection of three samples per vine. The numbers of ‘Zesy002′ samples available were reduced owing to losses of vines from Psa infection, and sample numbers were maintained by collecting paired samples from additional vines.

### 4.1. Non-Targeted LC-MS Analysis

Samples (100 mg DW or 500 mg FW) were extracted in 80% methanol (2.5 mL) or methanol (2 mL) respectively and diluted with methanol (5 fold) before analysis. At least one pair of biological replicate samples was included for each tissue harvested. Composite QC samples were measured interspersed after every tenth randomized analytical sample. The LC-MS system comprised a Dionex Ultimate^®^ 3000 Rapid Separation LC and a micrOTOF QII high resolution mass spectrometer (Bruker Daltonics, Bremen, Germany). The LC column was either a Hypersil GOLD C18 100 × 2.1 mm, 1.9 µm (Thermo Scientific, for ‘Hayward’) or a Luna Omega C18 100 × 2.1 mm, 1.6 µm (Phenomenex, for ‘Zesy002’) maintained at 40 °C, flow 400 µL/min. Solvents were A 0.2% formic acid and B 100% acetonitrile with a gradient of 90% A 0–0.5 min; linear gradient to 60% A, 0.5–7 min; linear gradient to 5% A, 7–12 min; held at 5% A, 12–15 min; linear gradient to 90% A, 15–15.2 min to return to the initial conditions before another sample injection at 18 min. The injection volume was 1 μL. Negative ion electrospray mass spectral scans were acquired at 5 scans/s with a capillary voltage of +3500 V: temperature 225 °C; drying N2 flow 6 L/min; nebulizer N2 1.5 bar, endplate offset –500 V, mass range 100–1500 Da. 

Instrument data files were processed using QuantAnalysis and MetaboScape 4.0 (Bruker Daltonics, Bremen, Germany) using standard software settings with de-replication of pseudomolecular and dimer ions, and water and formic acid adducts. The frequency of background ions was estimated from blank injections. Fourteen weak background ions were detected (Amax < 11,000 counts) compared with 295 metabolites retained in properly injected samples after data processing. Background ions were not removed from the dataset. Retention time stability was assessed by visual inspection of the overlaid chromatograms of the composite QC samples and was sufficient to allow alignment of all data by the MetaboScape software. Principal components analysis (PCA) showed close clustering of QC samples, with plant samples clustering by tissue type and no indication of run effects.

Analytical data were exported to Microsoft^®^ Excel before statistical analysis. For each sample type, all metabolites with a detection rate of <80% were removed. Four datasets (‘Zesy002’ H3 leaf, internode and fruit, and ‘Hayward’ H3 Leaf) contained over 1000 putative metabolites and this number was reduced by excluding weaker metabolic signals (Amax < 800 counts for the ‘Hayward’ H3 Leaf QC samples, <1750 area counts for ‘Zesy002’ leaf samples). The remaining MS signals are referred to as ‘metabolites’ for the purpose of this study. Single analyses resulting from injection failure or loss of vines due to Psa infection were removed. Data were normalized to sample dry weight (or fresh weight for fruit) and summary statistics were calculated of the numbers and percentages of missed metabolites, the mean, median and distributions of CV values, and numbers and percentage of metabolites with CV values below arbitrary thresholds.

Linear mixed effects models (REML procedure, Genstat version 19, VSNi Ltd., Hemel Hempstead, UK) were used to calculate the within-vine and between-vine components of variance for each metabolite for each tissue at each sampling time, and what proportion of the total variability was due to between-vine (inter-vine) variation. The effects of the sampler, whether the vine had Psa symptoms or not, and (for ‘Zesy002’) the effect of different rootstocks, were tested by fitting these parameters in the mixed effect models and testing whether removing these parameters made the model significantly worse. The mixed models analyzed individual metabolites. To look at whether inter-vine differences, Psa symptoms, sampler and (for ‘Zesy002’) rootstock influenced the pattern of metabolites, a multivariate ANOVA-like technique (Adonis, from the R package vegan) was used. For Adonis, data were log transformed, and Euclidean distance used.

### 4.2. Targeted Chemical Analysis

Phytohormones were quantified against their corresponding deuterated internal standards using a 5500 QTrap triple quadrupole/linear ion trap and sample clean-up protocols as reported [51]. Samples for soluble carbohydrate analysis (50 mg DW or 200 mg FW fruit) were extracted into 80% ethanol with fucose as internal standard and analyzed against known standards using a DIONEX ICS-5000 Reagent-Free™ IC (RFIC™) system [52]. Linear mixed effects models (Genstat REML procedure) were used to calculated within-vine and between-vine components of variance as above.

## Figures and Tables

**Table 1:** Technical replicates: Number of metabolites (mass tags) measured, median %CV and percentage of metabolites with CV < 20% measured in duplicate analyses of powdered or frozen samples from ‘Hayward’ and ‘Zesy002’ kiwifruit vines.

Harvest	Cultivar	Tissue	Number of Metabolites	Median CV	Percentage Metabolites with CV ≤ 20%
1	‘Hayward’	Internode	191	13.1	68
1	‘Hayward’	Mature leaf	282	9.3	73
1	‘Hayward’	Young leaf	- ^1^	-	-
2	‘Hayward’	Internode	183	7.0	86
2	‘Hayward’	Mature leaf	254	8.6	66
2	‘Hayward’	Fruit	188	9.4	77
3	‘Hayward’	Internode	630	7.2	86
3	‘Hayward’	Mature leaf	350	11.3	79
3	‘Hayward’	Fruit	438	8.3	86
1	‘Zesy002’	Internode	220	5.7	94
1	‘Zesy002’	Mature leaf	219	6.3	92
1	‘Zesy002’	Young leaf	234	7.0	94
2	‘Zesy002’	Internode	-	-	-
2	‘Zesy002’	Mature leaf	326	8.9	90
2	‘Zesy002’	Fruit	205	9.9	79
3	‘Zesy002’	Internode	274	11.9	82
3	‘Zesy002’	Mature leaf	878	6.0	95
3	‘Zesy002’	Fruit	335	6.6	98

^1^ Insufficient sample or one sample lost.

**Table 2:** Numbers of metabolites (mass tags) measured, and numbers and percentages of metabolites with CV less than 20%, measured in ‘Hayward’ and ‘Zesy002’ internode, and mature leaf issue and fruit samples at three harvest times.

Harvest	Cultivar	Tissue	Number of Metabolites	Number of Metabolites with CV ≤ 20%	Percentage Metabolites with CV ≤ 20%
1	‘Hayward’	Internode	460	95	21%
1	‘Hayward’	Mature leaf	510	117	23%
1	‘Hayward’	Young leaf	518	247	48%
2	‘Hayward’	Internode	447	138	31%
2	‘Hayward’	Mature leaf	489	123	25%
2	‘Hayward’	Fruit	404	87	22%
3	‘Hayward’	Internode	611	298	49%
3	‘Hayward’	Mature leaf	359	45	13%
3	‘Hayward’	Fruit	440	190	43%
1	‘Zesy002’	Internode	221	64	29%
1	‘Zesy002’	Mature leaf	228	86	38%
1	‘Zesy002’	Young leaf	228	87	38%
2	‘Zesy002’	Internode	295	79	27%
2	‘Zesy002’	Mature leaf	325	140	43%
2	‘Zesy002’	Fruit	202	39	19%
3	‘Zesy002’	Internode	531	219	41%
3	‘Zesy002’	Mature leaf	912	350	38%
3	‘Zesy002’	Fruit	350	181	52%

**Table 3:** Comparison of results of ANOVA and Adonis analysis of *Pseudomonas syringae* var. *actinidiae* (Psa) infection of vegetative tissue, sampler, inter-vine variation and rootstock on variability of metabolomics analysis of ‘Hayward’ kiwifruit tissues collected at three harvest times. Percentages in bold show more significant effects than expected by chance (ANOVA) or *p* < 0.05 (Adonis).

Harvest	Tissue	Number Metabolites CV < 20%	Mixed Models % Metabolites with Significant Effect (Adjusted for Other Factors)			Adonis *p*-Value (Adjusted for Other Factors)		
			Psa	Sampler	Vine	Psa	Sampler	Vine
1	Internode	95	0	1	3	0.32	0.86	0.50
1	Mature leaf	117	2	3	**7%** ^1^	0.07	0.29	0.09
1	Young leaf	247	**4%** ^1^	**12%** ^1^	**7%** ^1^	0.21	0.005	0.06
2	Fruit	87	0	2	1	0.56	0.001	0.08
2	Internode	138	1	0	3	0.25	0.16	0.22
2	Mature leaf	123	2	2	2	0.27	0.36	0.36
3	Fruit	190	1	3	3	0.49	0.04	0.014
3	Internode	298	1	1	2	0.33	0.70	0.007
3	Mature leaf	45	0	2	2	0.68	0.13	0.45

^1^ Number of significant effects more than be expected by chance, *p* = 0.01.

**Table 4:** Comparison of results from ANOVA and Adonis analysis of effects of *Pseudomonas syringae* var. *actinidiae* (Psa), sampler, between vine variation and rootstock on variability of metabolomics analysis of ‘Zesy002’ kiwifruit tissues collected at three harvest times. Values in bold show more significant effects than expected by chance (ANOVA) or *p* < 0.05 (Adonis).

Harvest	Tissue	Number Metabolites CV < 20%	Mixed Models % Metabolites with Significant Effect (Adjusted for Other Factors)				Adonis *p*-Value (Adjusted for Other Factors)			
			Psa	Sampler	Vine	Rootstock	Psa	Sampler	Vine	Rootstock
1	Internode	64	2	2	- ^1^	3	0.76	0.33	- ^1^	0.84
1	Mature leaf	86	2	0	0	1	0.37	0.11	0.72	0.70
1	Young leaf	87	1	0	1	0	0.75	0.21	0.26	0.50
2	Fruit	39	5	5	**8** ^2^	5	**0.014**	0.48	0.47	0.31
2	Internode	79	0	0	0	**18** ^2^	**0.001**	0.09	0.05	**0.003**
2	Mature leaf	140	1	1	1	**7** ^2^	0.06	0.49	**0.02**	**0.001**
3	Fruit	181	- ^1^	0	1	**5** ^2^	- ^1^	**0.02**	0.07	0.10
3	Internode	219	-	0	1	**4** ^2^	-	0.19	0.86	0.06
3	Mature leaf	350	-	0	2	2	-	0.67	0.44	0.10

^1^ Insufficient replicates or absence of Psa symptoms on vegetative tissue; ^2^ percent more than expected by chance, *p* = 0.01.

**Table 5:** Mean (and range) %CV and mean %CV due to between-vine variation for soluble sugars and polyols measured, leaf and fruit of ‘Hayward’ and ‘Zesy002’ kiwifruit at three harvest times, and for nine gibberellins, cytokinins and stress phytohormones where these co-occur in selected plant tissues.

	Soluble Sugars and Polyols		Phytohormones	
	‘Hayward’	‘Zesy002’	‘Hayward’	‘Zesy002’
%CV (mean and rage)	35.1 (11–152)	36.1 (9–93)	42.9 (10–104)	47.0 (11–131)
% between-vine variation (mean and rage)	22.6 (0–70)	35.0 (0–96)	27.5 (0–94)	43.9 (0–93)

**Table 6:** Numbers of samples collected from each *Actinidia* cultivar. Fruit samples are in italics.

Cultivar	‘Hayward’			‘Zesy002’		
	Internode	Mature Leaf	Young Leaf (*Fruit*)	Internode	Mature Leaf	Young Leaf (*Fruit*)
Harvest 1	28	27	29	18	19	23
Harvest 2	29	30	(*28*)	21	23	(*23*)
Harvest 3	29	29	(*29*)	18	19	(19)

## Data Availability

The data presented in this study are available in the Supplementary Material.

## Supplementary Materials

The following are available online at https://www.mdpi.com/article/10.3390/metabo11090603/s1, Table S1: Metabolite variability in QC composite samples, Table S2: Median concentrations (mg/g), %CV and %CV due inter-vine variation of soluble carbohydrates measured in internode, leaf and fruit of Hayward kiwifruit at three harvest times, Table S3: Median concentrations (mg/g), %CV and %CV due inter-vine variation of soluble carbohydrates measured in internode, leaf and fruit of Gold3 kiwifruit at three harvest times, Table S4: Median concentrations (ng/g), %CV and %CV due inter-vine variation of selected phytohormones measured in internode, leaf and fruit of Hayward and Gold3 kiwifruit at three harvest times, Figure S1: Principal Components Analysis of Psa, sampler and individual vine effects on metabolite profiles in internode, leaf and fruit tissues of ‘Hayward’ kiwifruit collected at three harvest times. Data are for metabolites with CV < 20% in the experimental data, Figure S2: Principal Components Analysis of Psa, sampler and individual vine effects on metabolite profiles in internode, leaf and fruit tissues of Gold3 kiwifruit collected at three harvest times. Data are for metabolites with CV < 20% in the experimental data. Datafiles: ‘HaywardmetabolomicsdataallANOVA.xlsx’, ‘Zesy002metabolomicdataallANNOVA.xlsx’.
