# Supplementary material for: Kiwifruit Metabolomics—An Investigation of within Orchard Metabolite Variability of Two Cultivars of Actinidia chinensis
Source: Metabolites. 2021 Sep 6;11(9):603. doi: 10.3390/metabo11090603 (PMC8468816; doi:10.3390/metabo11090603)
Supplement: Supplementary file 1 [file metabolites-11-00603-s001.zip › metabolites-1362790-supplementary/metabolites-1362790 - Supplementary Figures and Tables.pdf]

Supplementary Materials

Table S1. Metabolite variability in QC composite samples

Table S2. Median concentrations (mg/g), %CV and %CV due inter-vine variation of soluble carbohydrates measured in internode, leaf and fruit of Hayward kiwifruit at three harvest times.

Table S3. Table S3. Median concentrations (mg/g), %CV and %CV due inter-vine variation of soluble carbohydrates measured in internode, leaf and fruit of Zesy002 kiwifruit at three harvest times.

Table S4. Median concentrations (ng/g), %CV and %CV due inter-vine variation of selected phytohormones measured in internode, leaf and fruit of Hayward and Zesy002 kiwifruit at three harvest times.

Figure S1. Principal Components Analysis of Psa, sampler and individual vine effects on metabolite profiles in internode, leaf and fruit tissues of 'Hayward' kiwifruit collected at three harvest times. Data are for metabolites with CV <20% in the experimental data.

Figure S2. Principal Components Analysis of Psa, sampler and individual vine effects on metabolite profiles in internode, leaf and fruit tissues of Zesy002 kiwifruit collected at three harvest times. Data are for metabolites with CV <20% in the experimental data.

Datafiles:

Hayward metabolomics data: 'HaywardmetabolomicsdataallANOVA.xlsx'.

Zesy002 metabolomics data: 'Zesy002metabolomicdataallANNOVA.xlsx'.

Table S1. Metabolites (mass tags) measured and percent with CV<20% (bracketed) in composite QC samples (typically n = 6) of 'Hayward' and Zesy002 kiwifruit vines.

Cultivar	'Hayward'			Zesy002		
Tissue	Internode	*Mature leaf*	Young leaf/*fruit*	Internode	*Mature leaf*	Young leaf/*fruit*
Harvest 1	392 (91)	*458 (92)*	*503 (94)*	276 (92)*	276 (92)*	276 (92)*
Harvest 2	376 (91)	*450 (94)*	***365** (90)*	362 (99)*	362 (99)*	362 (99)*
Harvest 3	615 (92)	*606 (94)*	***448** (85)*	521 (90)	*629(95)*	***375** (83)*

* Composite sample prepared from internode, and mature and young leaf Gold3 tissue samples.

Table S2. Median concentrations (mg/g), %CV and %CV due inter-vine variation of soluble carbohydrates measured in internode, leaf and fruit of Hayward kiwifruit at three harvest times. Concentrations are mg/g fresh weight (FW) or mg/g dry weight (DW).

**Hayward kiwifruit**		**Harvest 1**			**Harvest 2**			**Harvest 3**			**Mean**	**Range**
		**Internode**	**Mature leaf**	**Young leaf**	**Internode**	**Mature leaf**	**Fruit**	**Internode**	**Mature leaf**	**Fruit**		
		DW	DW	DW	DW	DW	FW	DW	DW	FW		
***myo*-Inositol**	**median concentration**	**7.4**	**8.2**	**8.1**	**2.1**	**8.5**	**0.9**	**4.4**	**12.1**	**1.5**		
	%CV of samples	22	25	35	18	26	11	27	19	12	21.7	11 - 35
	%CV due inter-vine variability	0	66	9	31	29	16	51	0	59	29.0	0 - 66
**Galactinol**	**median concentration**	**1.7**	**2.3**	**2.1**	**0.8**	**2**	**0.1**	**1**	**2.4**	**0.1**		
	%CV of samples	17	29	40	20	25	32	26	21	20	25.6	17 - 40
	%CV due inter-vine variability	17	10	43	8	0	7	20	4	34	15.9	0 - 43
**Glucose**	**median concentration**	**20.6**	**20.5**	**11.9**	**0.7**	**2**	**2.8**	**0.2**	**2**	**2**		
	%CV of samples	28	35	47	84	70	24	148	47	36	57.7	24 - 148
	%CV due inter-vine variability	0	46	0	17	1	70	1	37	28	22.2	0 - 70
**Fructose**	**median concentration**	3.4	7.5	4	0.7	3.1	0.9	0.3	2.7	0.5		
	%CV of samples	40	35	42	73	57	18	152	58	33	56.4	18 - 152
	%CV due inter-vine variability	33	44	55	19	0	35	5	70	49	34.4	0 - 70
**Sucrose**	**median concentration**	**27.4**	**35.3**	**29.2**	**14.1**	**33.7**	**1**	**19.8**	**44.2**	**0.8**		
	%CV of samples	14	13	24	14	19	26	20	17	17	18.2	13 - 26
	%CV due inter-vine variability	5	0	41	15	0	27	9	0	25	13.6	0 - 41
**Planteose**	**median concentration**	**1.1**	**1.2**	**2**	**1.9**	**12.7**	**0.1**	**4**	**10.3**	**0.1**		
	%CV of samples	22	30	27	25	25	34	27	33	59	31.3	22 - 59
	%CV due inter-vine variability	31	15	2	69	0	39	9	0	17	20.2	0 - 69

Table S3. Median concentrations (mg/g), %CV and %CV due inter-vine variation of soluble carbohydrates measured in internode, leaf and fruit of Zesy002 kiwifruit at three harvest times. Concentrations are mg/g fresh weight (FW) or mg/g dry weight (DW). 'nd' below LOD taken as 5 X S/N

**Gold3 Kiwifruit**		**Harvest 1**			**Harvest 2**			**Harvest 3**			**Mean**	**Range**
		**internode**	**mature leaf**	**young leaf**	**internode**	**mature leaf**	**fruit**	**internode**	**mature leaf**	**fruit**		
		DW	DW	DW	DW	DW	FW	DW	DW	FW		
***myo*-Inositol**	**median concentration**	**4.6**	**8.8**	**8.5**	**3.2**	**8.7**	**1.5**	**2.5**	**7.1**	**1.1**		
	%CV of samples	44	17	15	26	15	35	19	19	14	22.7	14 - 44
	%CV due inter-vine variability	71	35	43	24	52	88	0	59	96	52.0	0 - 96
**Galactinol**	**median concentration**	**1**	**1.5**	**1.7**	**0.5**	**2.5**	**0**	**0.6**	**2.5**	**0.1**		
	%CV of samples	30	34	21	23	38	36	24	33	15	28.2	15 - 38
	%CV due inter-vine variability	67	65	0	41	80	14	0	13	39	35.4	0 - 80
**Glucose**	**median concentration**	**9**	**19**	**9.9**	**0.3**	**5.7**	**6.6**	**0.3**	**3.2**	**2.7**		
	%CV of samples	75	33	21	87	70	9	71	32	25	47.0	9 - 87
	%CV due inter-vine variability	19	3	28	58	0	0	51	38	54	27.9	0 - 56
**Fructose**	**median concentration**	5.6	21.9	3.5	0.5	9.5	2.9	0.5	3.8	2.4		
	%CV of samples	70	37	45	86	62	29	70	45	24	52.0	24 - 86
	%CV due inter-vine variability	4	67	19	72	0	62	0	30	54	34.2	0 - 72
**Sucrose**	**median concentration**	**56.9**	**63.7**	**19.1**	**20.5**	**37.2**	**0.8**	**11.9**	**27**	**1**		
	%CV of samples	15	26	15	18	21	74	13	20	12	23.8	12 - 74
	%CV due inter-vine variability	44	72	0	45	53	91	0	34	65	44.9	0 - 91
**Planteose**	**median concentration**	**nd**	**9**	**0.7**	**2.7**	**16.8**	**nd**	**4.3**	**10.2**	**0.1**		
	%CV of samples		53	93	32	38		25	43	18	43.1	18 - 93
	%CV due inter-vine variability		6	0	60	4		39	0	0	15.6	0 - 60

Table S4. Median concentrations (ng/g), analytical %CV calculated from technical replicates and %CV due inter-vine variation of selected phytohormones measured in internode, leaf and fruit of Hayward and Zesy002 kiwifruit at three harvest times. Concentrations are expressed as either ng/g fresh weight (FW) or ng/g dry weight (DW). 'nd' samples are below LOD taken as 5 X S/N. GA1, GA4 gibberellins 1 and 4, 2iP *N*6-isopentylenyladenine, 2iPR *N*6-isopentylenyladenosine, tZ *trans*-zeatin, tZR *trans*-zeatin riboside, SA salicylic acid, ABA abscisic acid, JA jasmonic acid. Quantitation against deuterated internal standards using targeted LC-MS/MS.

		**Hayward**				**Zesy002**				
		Young Leaf Harvest 1 DW	Internode Harvest 1 DW	Fruit Harvest 2 FW	Fruit Harvest 3 FW	Young Leaf Harvest 1 DW	Internode Harvest 1 DW	Fruit Harvest 2 FW	Internode Harvest 2 DW	Fruit Harvest 3 FW
**GA1**	**median concentration**	**3.2**	**nd**	**0.7**	**0.5**	**1.3**	**1.5**	**0.1**	**0.9**	**nd**
	analytical CV (%)	3.8	-	3.7	15	5.7	-*	1.4	-	-
	%CV samples	32	-	27	35	52	43	46	66	-
	% CV due inter-vine variability	0	-	0	0	56	0	46	71	-
**GA4**	**median concentration**	**3.1**	**2.2**	**0.3**	**nd**	**3.5**	**2.1**	**nd**	**0.4**	**nd**
	analytical CV (%)	4.4	11	6.1		1.0	-*	-	7.3	-
	%CV samples	23	68	40		50	46	-	58	-
	% CV due inter-vine variability	44	0	0		1	0	-	60	-
**2iP**	**median concentration**	**1.0**	**4.7**	**nd**	**nd**	**1.4**	**7.9**	**0.1**	**3.9**	**0.2**
	analytical CV (%)	5.6	6.4	-	-	4.0	4.7	2.8	6.7	6.3
	%CV samples	15	55	-	-	88	28	36	36	23
	% CV due inter-vine variability	62	67	-	-	91	44	47	73	66
**2iPR**	**median concentration**	**8.3**	**95**	**0.6**	**nd**	**23.6**	**187.1**	**1.6**	**92.4**	**4.5**
	analytical CV (%)	1.3	6	8.3	-	4.9	5.3	1.9	3.4	5.4
	%CV samples	11	33	29	-	41	30	38	51	28
	% CV due inter-vine variability	0	0	58	-	71	29	67	80	0

**tZ**	**median concentration**	**1.6**	**2.6**	**nd**	**nd**	**2.7**	**3.0**	**0.1**	**1.1**	**0.3**
	analytical CV (%)	3.6	7.1	-	-	14.3	4.30	8.37	-	15.1
	%CV samples	24	104	-	-	97	48	131	36	38
	% CV due inter-vine variability	48	41	-	-	69	84	14	63	63
**tZR**	**median concentration**	**8.4**	**40**	**1.3**	**nd**	**12.1**	**28.0**	**1.8**	**20.0**	**6.3**
	analytical CV (%)	1.7	4.7	6.7	-	2.3	1.8	6.3	5.0	7.0
	%CV samples	27	54	35	-	24	44	51	58	46
	% CV due inter-vine variability	58	0	72	0	32	42	26	25	77
**SA**	**median concentration**	**2750**	**1255**	**117**	**38**	**710**	**240**	**18**	**80**	**19**
	analytical CV (%)	16	1.8	15	8	5.9	1.5	7.9	4.9	7.8
	%CV samples	33	29	67	57	28	27	11	15	23
	% CV due inter-vine variability	47	9	36	10	18	67	0	52	-
**ABA**	**median concentration**	**1197**	**1380**	**32**	**38**	**298**	**358**	**93**	**339**	**33**
	analytical CV (%)	2	2.8	3.4	0.9	5.5	2.1	3.1	1.6	3.4
	%CV samples	10	26	22	42	21	31	79	47	35
	% CV due inter-vine variability	10	33	41	94	0	11	93	65	67
**JA**	**median concentration**	**189**	**140**	**72**	**5.2**	**282**	**41**	**16**	**19**	**8.2**
	analytical CV (%)	1.3	17	10	3.7	7.4	10.7	6.1	2.8	6.6
	%CV samples	84	92	56	46	50	85	43	75	57
	% CV due inter-vine variability	1	0	28	0	54	93	11	0	31

* Insufficient duplicate or triplicate samples to calculate analytical CV.

Figure S1. Principal Components Analysis of Psa, sampler and individual vine effects on metabolite profiles in internode, leaf and fruit tissues of ‘Hayward’ kiwifruit collected at three harvest times. Data are for metabolites with CV <20% in the experimental data.

**‘Hayward’ Harvest 1**		
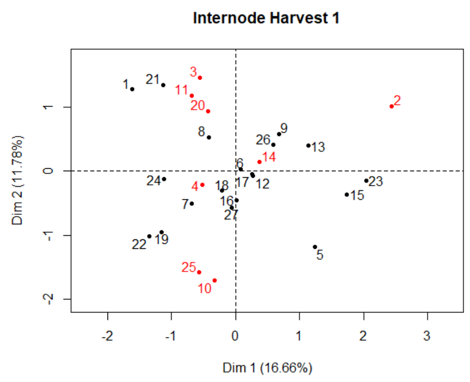	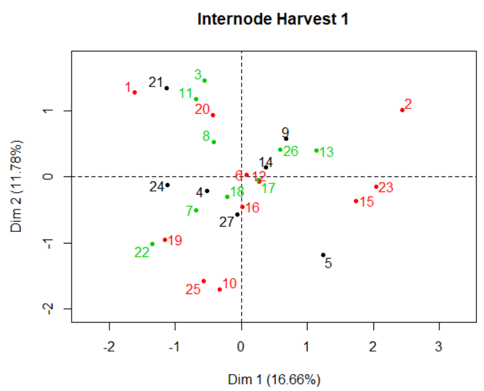	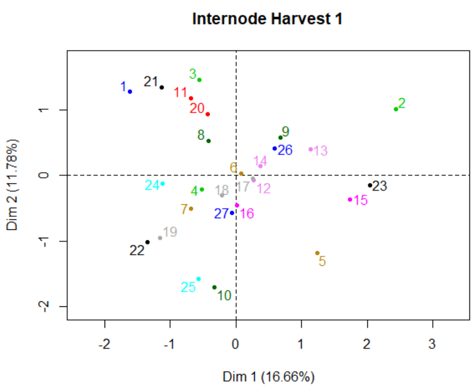
Psa symptoms (red), no symptoms (black)	Coloured by sampler	Coloured for individual vines
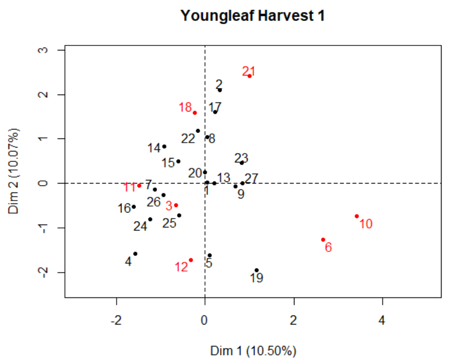	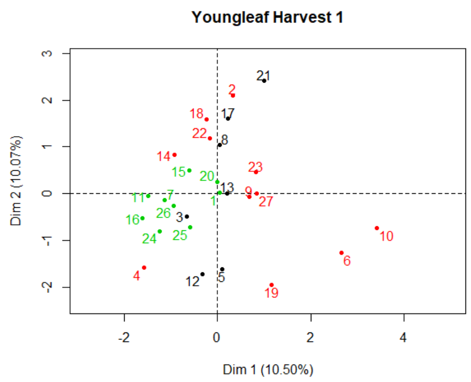	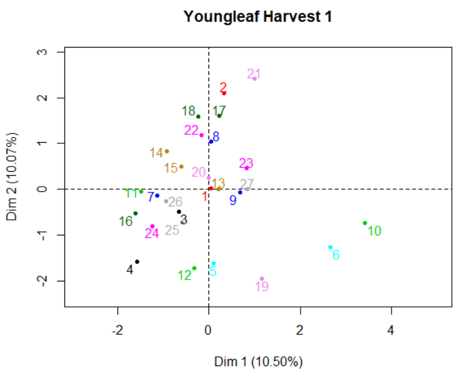
Psa symptoms (red), no symptoms (black)	Coloured by sampler	Coloured for individual vines

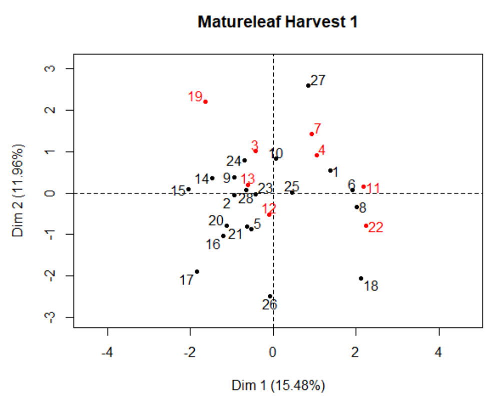	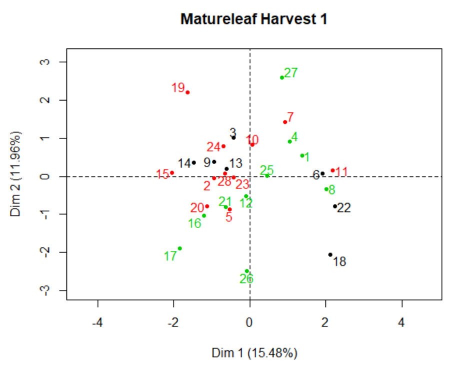	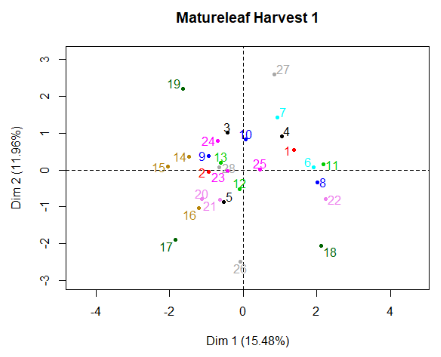
Psa symptoms (red), no symptoms (black)	Coloured by sampler	Coloured for individual vines

'Hayward' Harvest 2

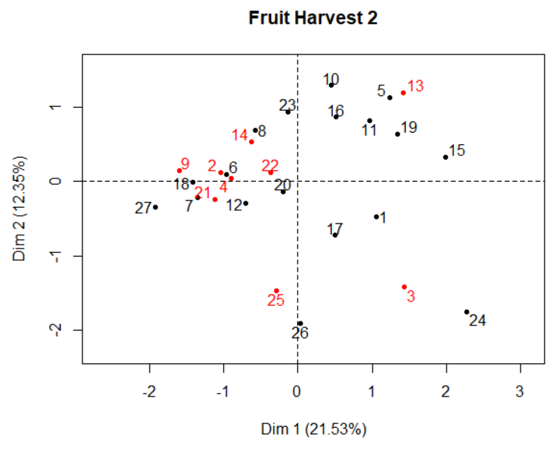	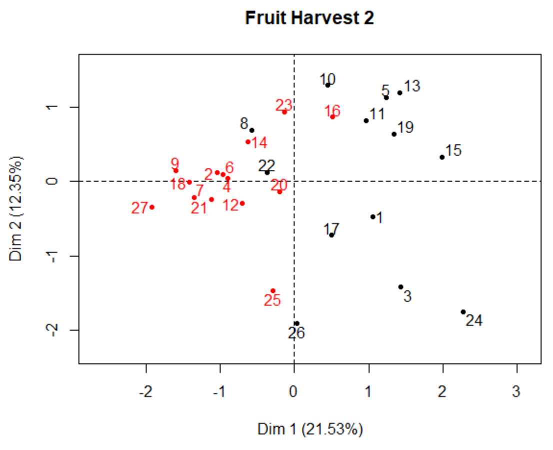	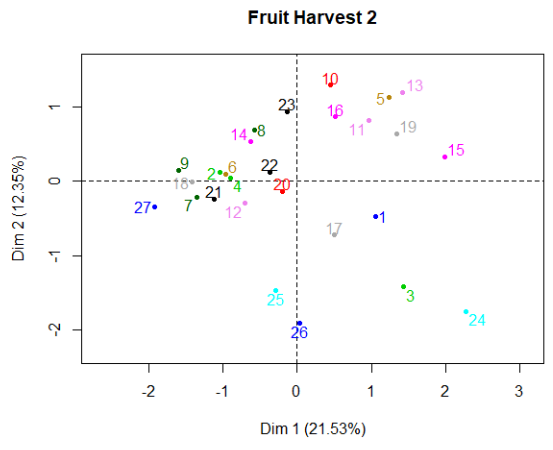
Psa symptoms (red), no symptoms (black)	Coloured by sampler	Coloured for individual vines

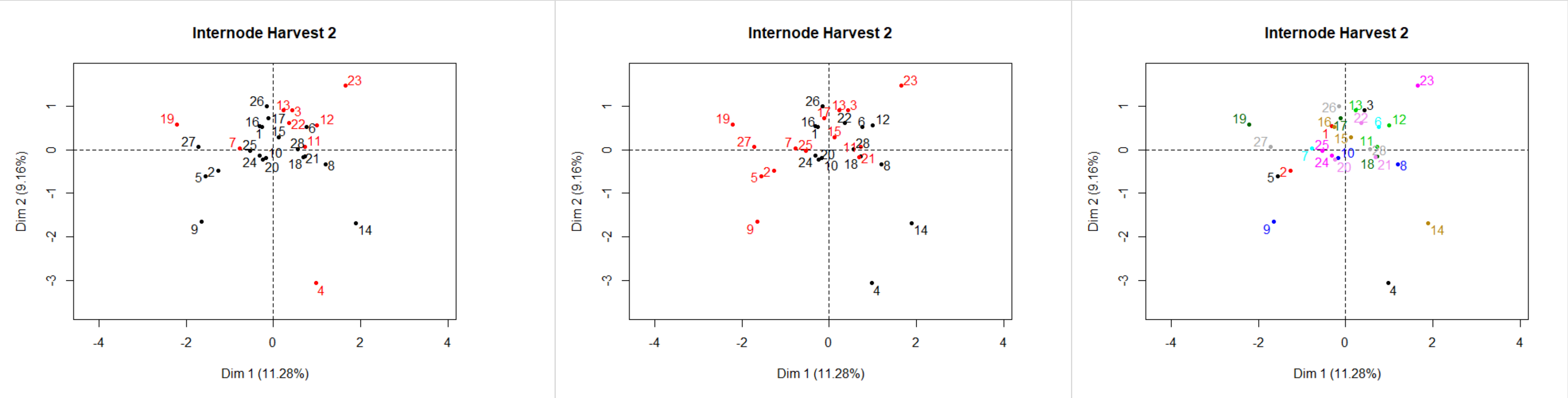

Psa symptoms (red), no symptoms (black)	Coloured by sampler	Coloured for individual vines

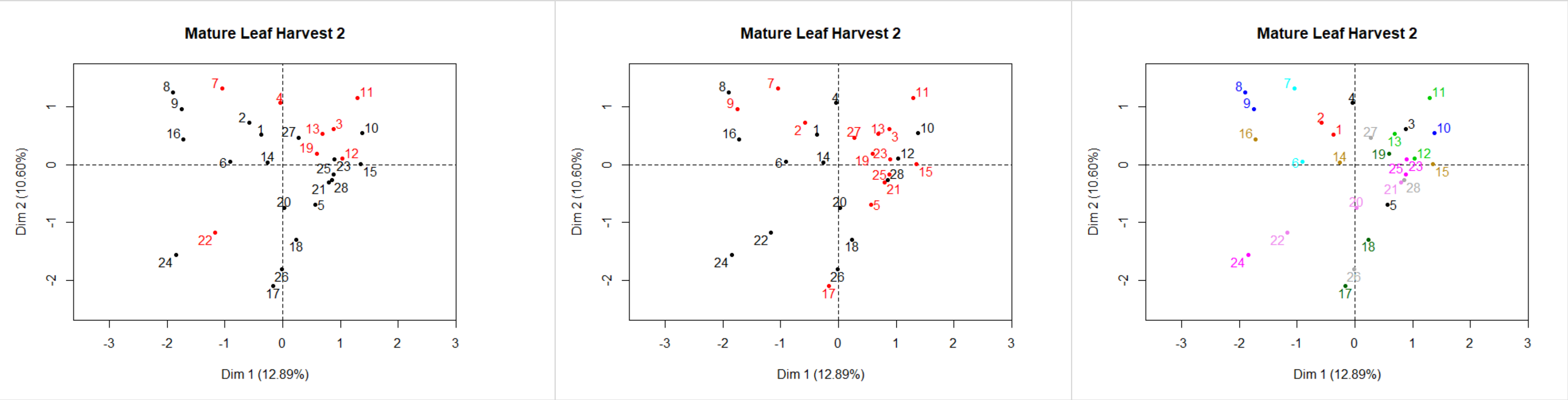

Psa symptoms (red), no symptoms (black)	Coloured by sampler	Coloured for individual vines

‘Hayward’ Harvest 3

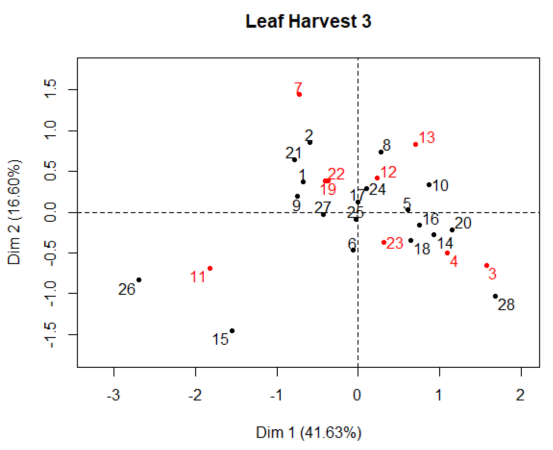

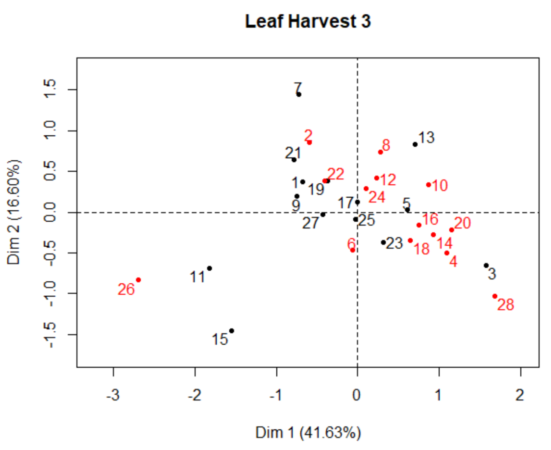

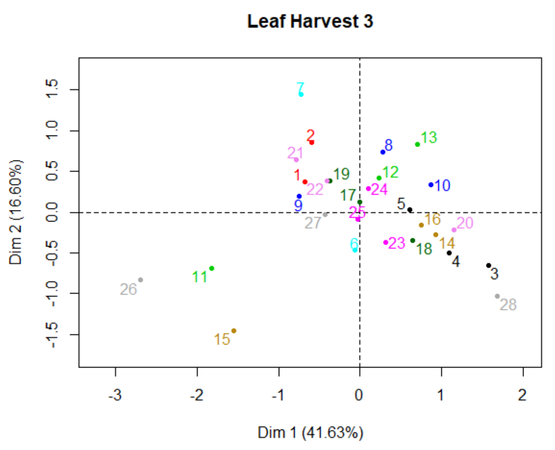

Psa symptoms (red), no symptoms (black)	Coloured by sampler	Coloured for individual vines

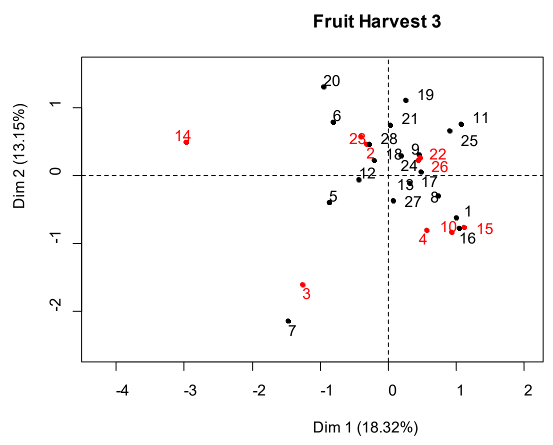

Psa symptoms (red), no symptoms (black)

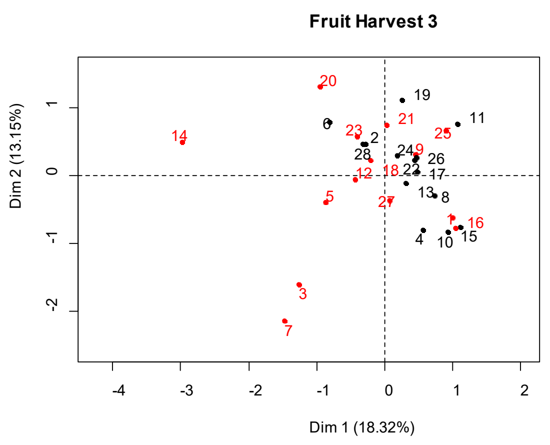

Coloured by sampler

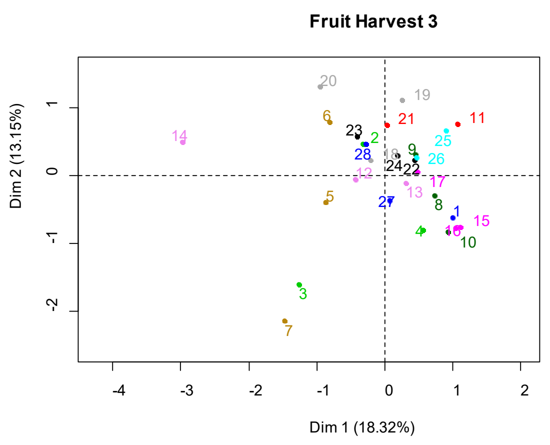

Coloured for individual vines

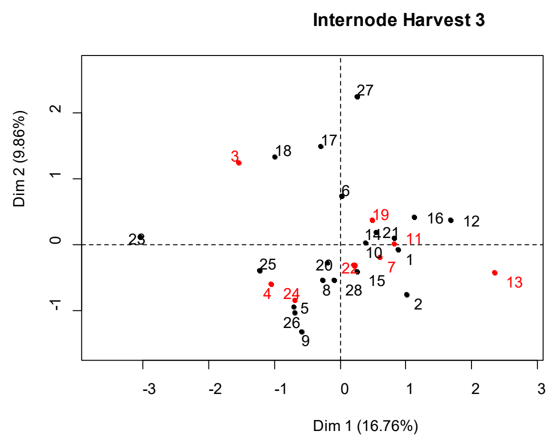

Psa symptoms (red), no symptoms (black)

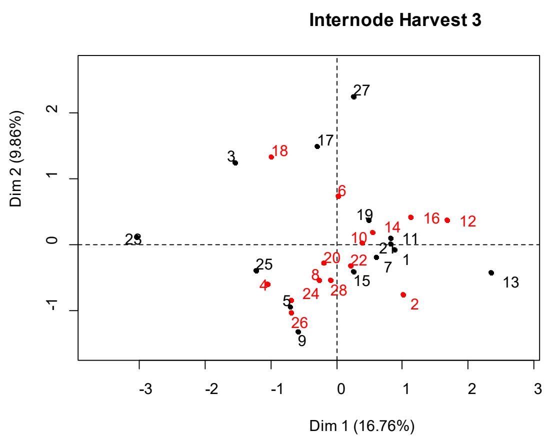

Coloured by sampler

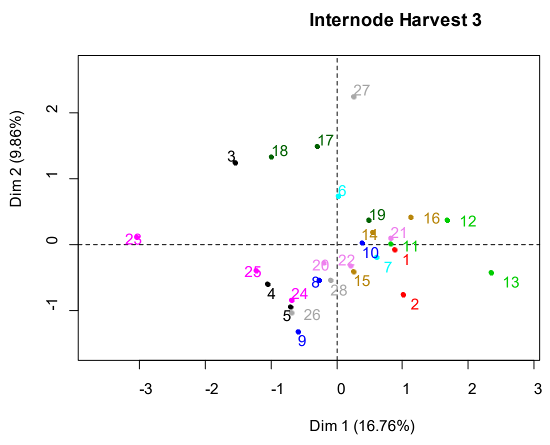

Coloured for individual vines

Figure S2. Principal Components Analysis of Psa, sampler and individual vine effects on metabolite profiles in internode, leaf and fruit tissues of Zesy002 kiwifruit collected at three harvest times. Data are for metabolites with CV <20% in the experimental data.

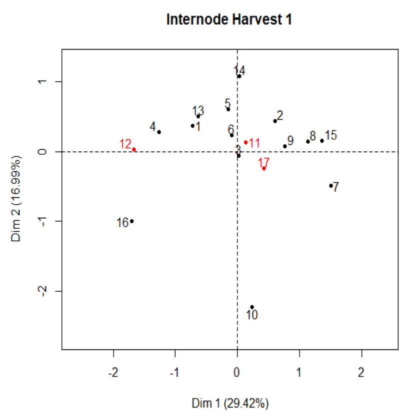

Psa symptoms (red), no symptoms (black)

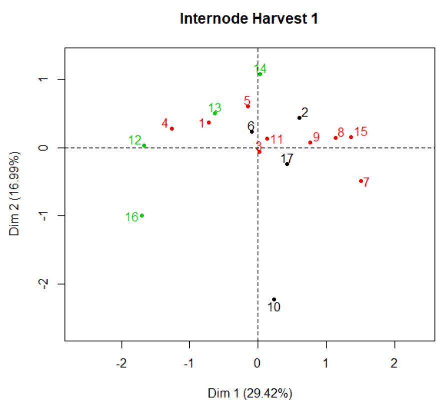

Coloured by sampler

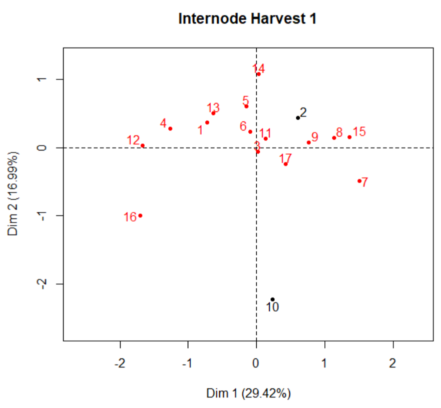

Stump grafted (red), 'Bruno' rootstock (black)

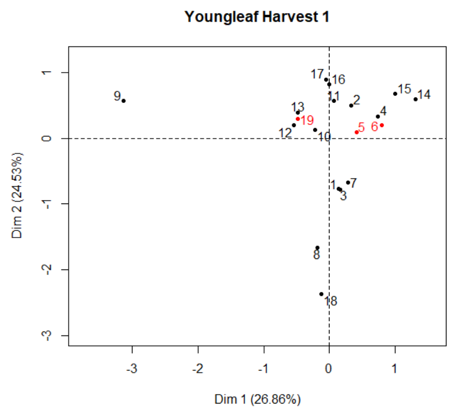

Psa symptoms (red), no symptoms (black)

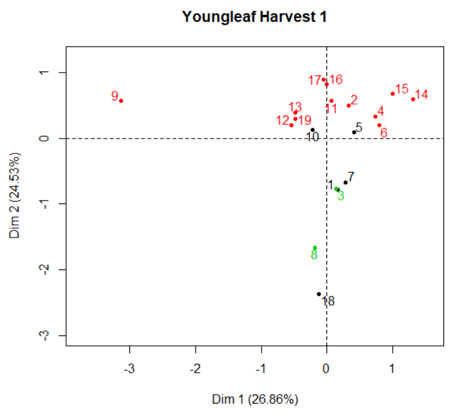

Coloured by sampler

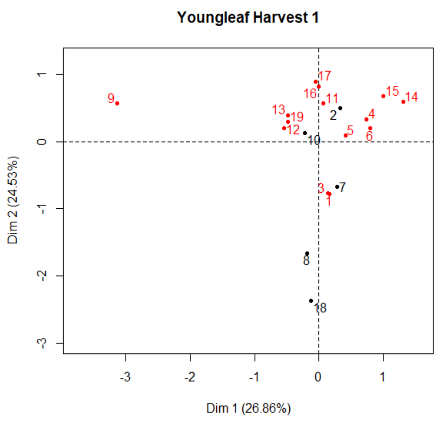

Stump grafted(red), 'Bruno' rootstock (black)

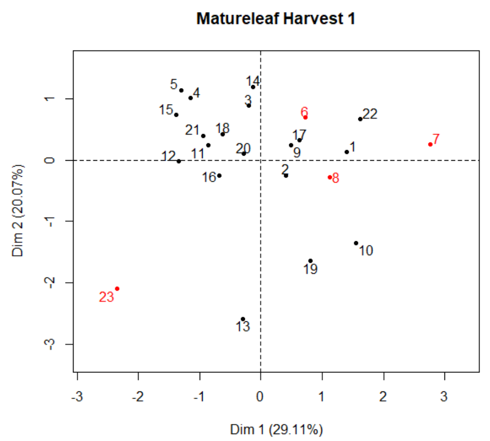

Psa symptoms (red), no symptoms (black)

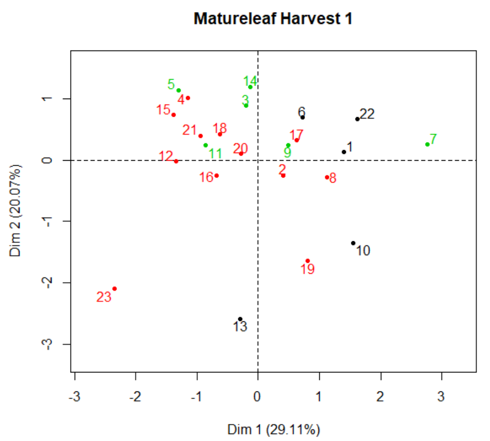

Coloured by sampler

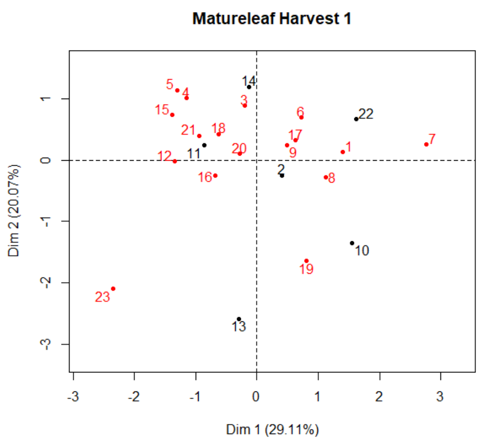

Stump grafted (red), 'Bruno' rootstock (black)

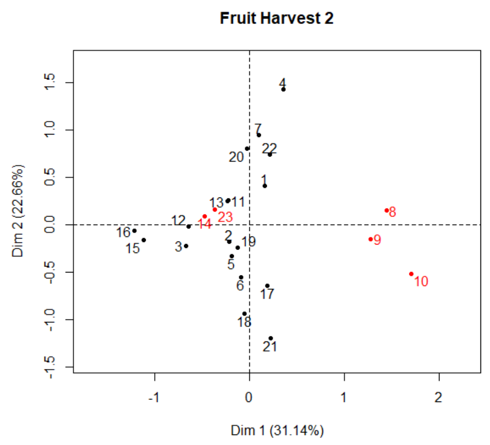

Psa symptoms (red), no symptoms (black)

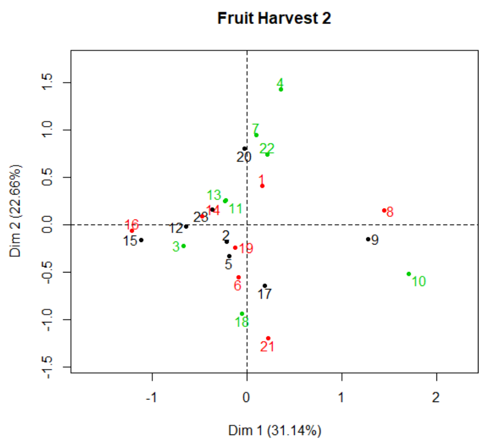

Coloured by sampler

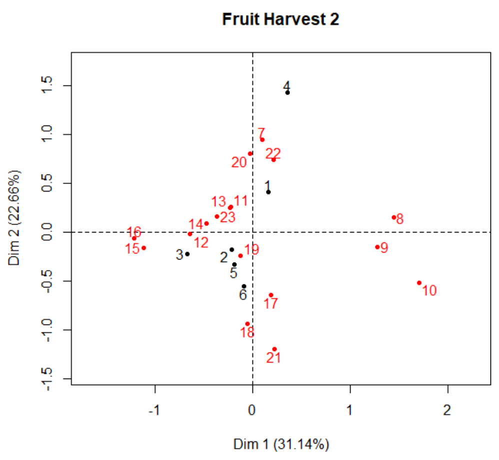

Stump grafted (red), 'Bruno' rootstock (black)

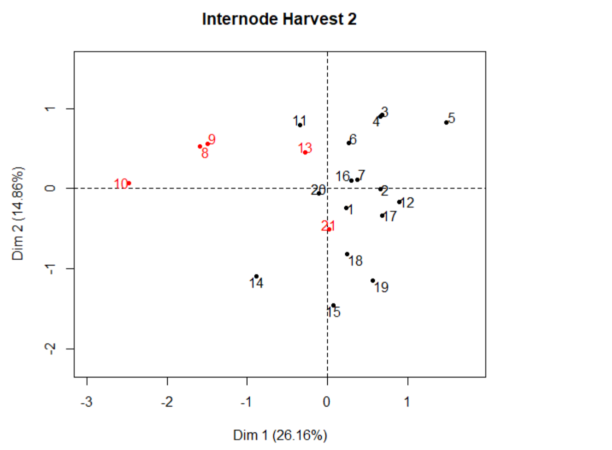

Psa symptoms (red), no symptoms (black)

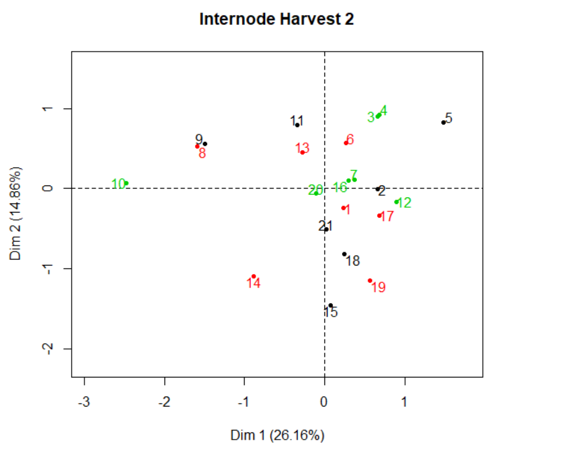

Coloured by sampler

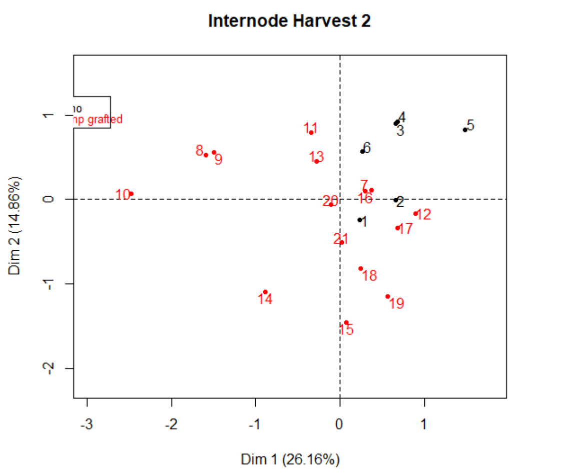

Stump grafted (red), 'Bruno' rootstock (black)

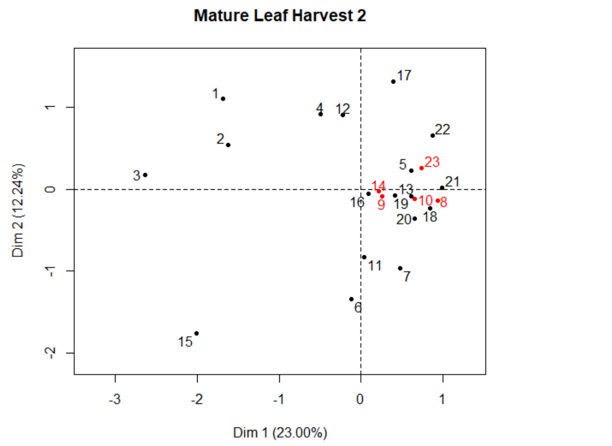

Psa symptoms (red), no symptoms (black)

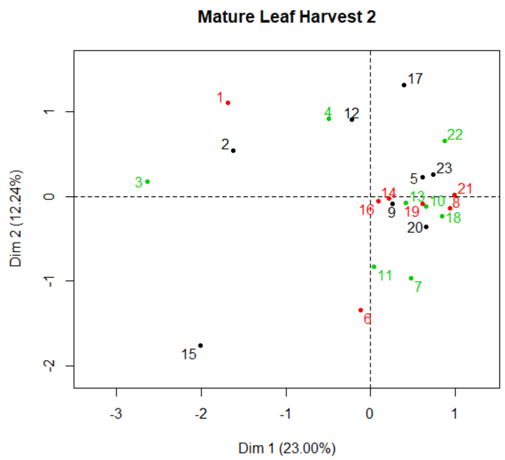

Coloured by sampler

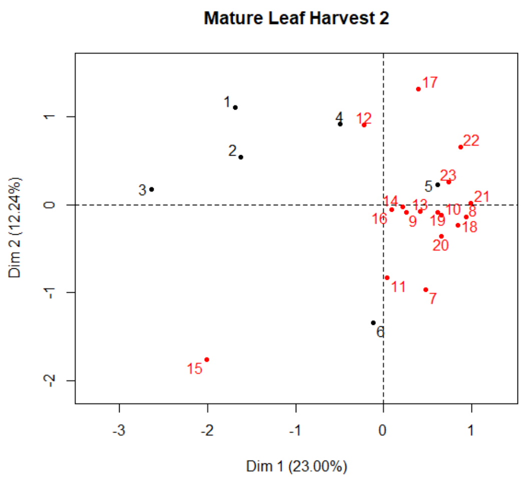

Stump grafted (red), 'Bruno' rootstock (black)

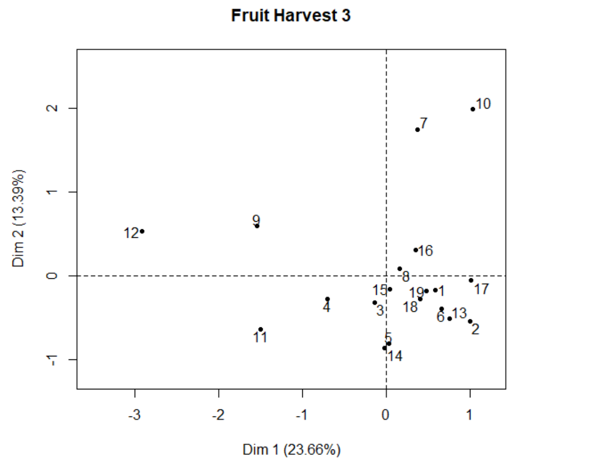

Psa symptoms (red), no symptoms (black)

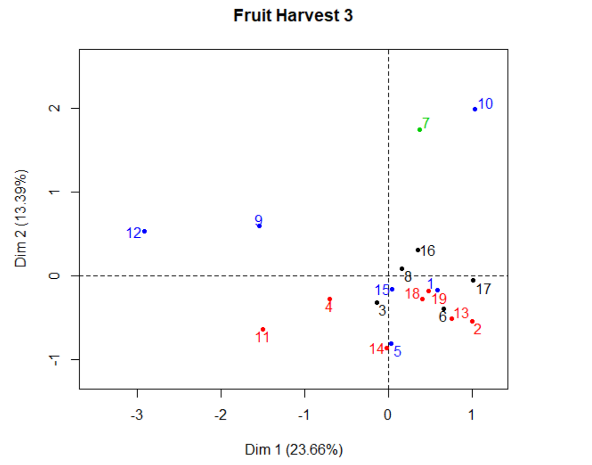

Coloured by sampler

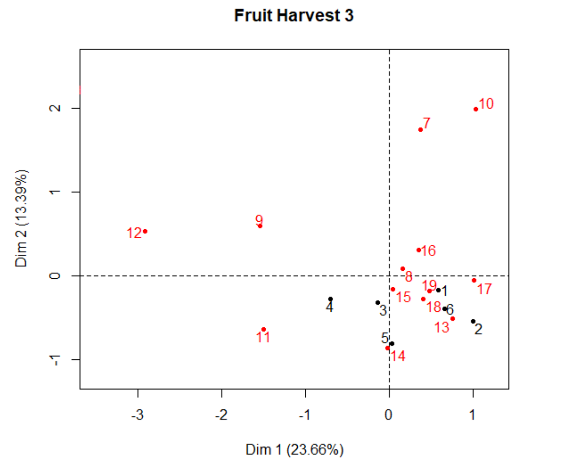

Stump grafted (red), 'Bruno' rootstock (black)

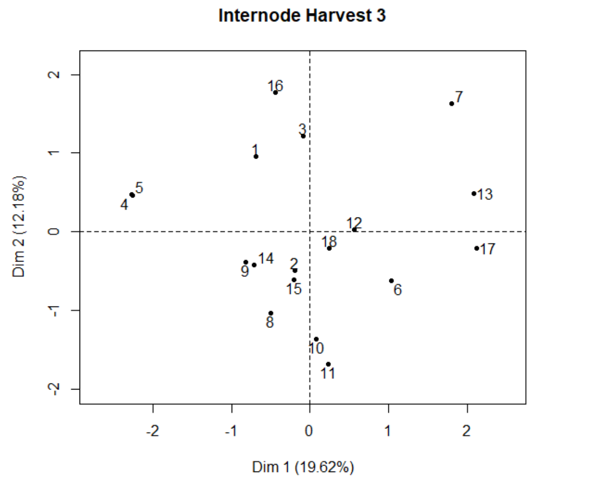

Psa symptoms (red), no symptoms (black)

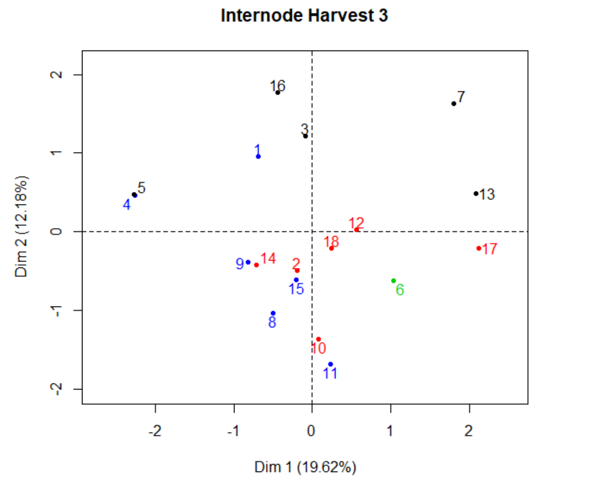

Coloured by sampler

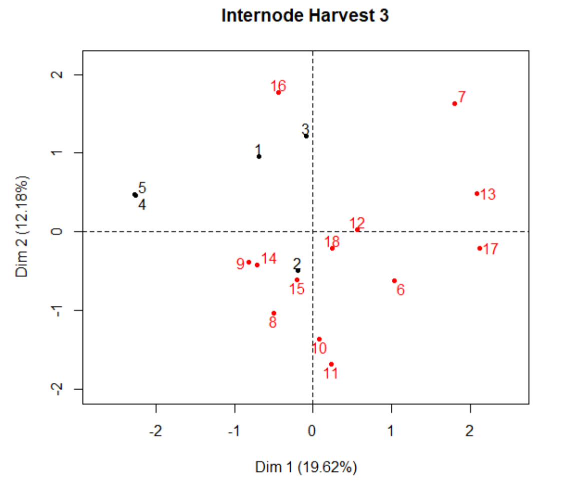

Stump grafted (red), 'Bruno' rootstock (black)

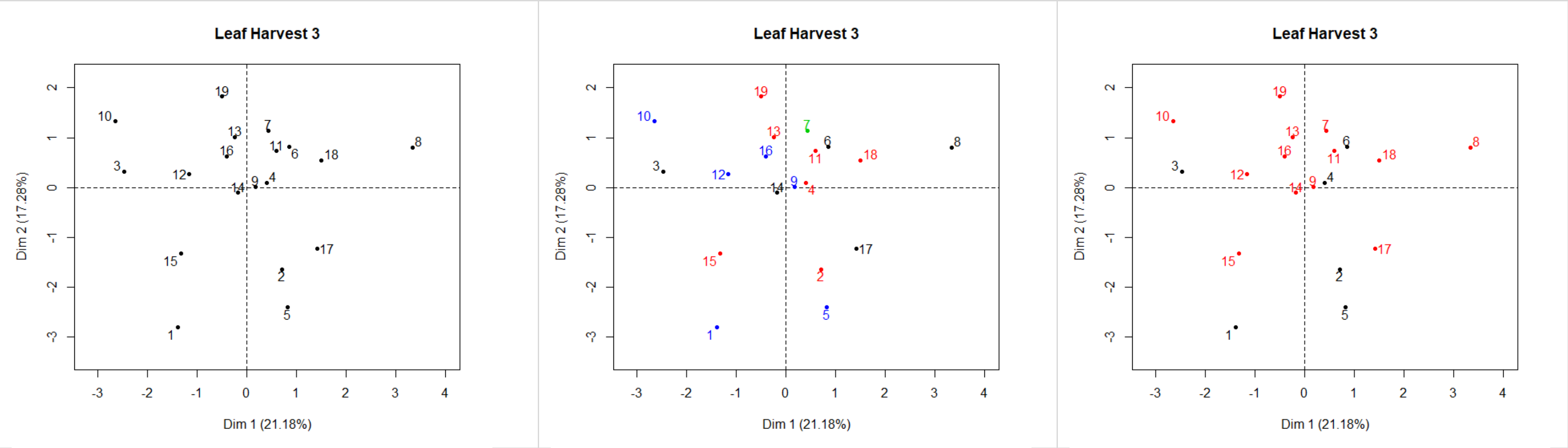

Psa symptoms (red), no symptoms (black)	Coloured by sampler	Stump grafted (red), ‘Bruno’ rootstock (black)
